# User satisfaction with child and adolescent mental health services: the association between user satisfaction and clinical outcomes

**DOI:** 10.1186/s12888-024-05715-1

**Published:** 2024-04-15

**Authors:** Mathiassen Børge, Arnesen Yngvild

**Affiliations:** 1 Department of Child and adolescent psychiatry, University Hospital of North Norway, Postboks 43, 9038 Tromsø, Norway; 2 Department of Psychology, UiT The Arctic University of Norway, Tromsø, Norway; 3 Department of Clinical Medicine, The Arctic University of Norway, Tromsø, Norway

**Keywords:** Child and adolescent mental health services, User satisfaction, Experience of service questionnaire, Outcome, Service improvement

## Abstract

**Background:**

This study aimed to examine the association between user satisfaction and clinical outcomes with child and adolescent mental health services (CAMHS) from the perspective of young people and their parents. The evidence bases for CAMHS user satisfaction measures are limited, with few studies investigating the link between user satisfaction and clinical outcomes. In particular, the perspectives of young people are missing.

**Methods:**

The parent and youth versions of the Experience of Service Questionnaire (ESQ), which evaluates the factors of general satisfaction (GS), satisfaction with care (SWC) and satisfaction with environment (SWE), were used to measure user satisfaction. The outcome measures were scores on the Strengths and Difficulties Questionnaire (SDQ), Children’s Global Assessment Scale (CGAS), and Health of the Nation Outcome Scales for Children and Adolescents (HoNOSCA). Hierarchical regression analysis was conducted on data collected from 233 young people and 495 parents who utilized CAMHS services.

**Results:**

GS and SWC predicted outcomes for both young people (ΔR^2^ = 0.08, *p* <.05) and parents (ΔR^2^ = 0.01, *p* <.05), indicating that user satisfaction had a significant impact on clinical outcomes for CAMHS users. In addition, GS and SWC significantly predicted young people-reported outcomes in the interaction model (ΔR^2^ = 0.10, *p* <.05), while no significant association was found with parent-reported outcomes (ΔR^2^ = 0.02, *p* =.09).

**Conclusion:**

User satisfaction, particularly for young people, has a significant impact on clinical outcomes. The causal relationship between user satisfaction and mental health outcomes requires further study.

## Background

Tracking patient experiences through user satisfaction measures is crucial to ensure children and young people can express opinions on matters affecting them. However, despite the call for routine tracking of user satisfaction for nearly four decades, it has yet to become standard for most services [1–3]. The lack of user satisfaction reports leaves a substantial gap in the ability to determine the quality of care provided [4, 5]. User satisfaction is a complex construct, and there have been mixed findings regarding its relationship with clinical outcomes and costs [5–8]. In the case of child and adolescent mental health services (CAMHS), there is limited research on the link between user satisfaction and clinical outcomes [1, 9].

Understanding user satisfaction with CAMHS is challenging due to the incongruity between feedback from young people and parents. Parent reports are more common in the literature [1, 9–13], but research indicates that young people’s feedback more accurately reflects the quality of care provided [14]. With a few exceptions [15], the literature indicates high satisfaction ratings for both YP and parents but weak correlations between the two [16–22]. Furthermore, parent satisfaction is typically assessed, but YP satisfaction is linked with better treatment outcomes in CAMHS [23]. Garland, Haine [19] found a cross-informant effect, showing that improved function reported by YP was associated with higher parent satisfaction. Although small in magnitude, the results of Turchik, Karpenko [16] also revealed improvements in clinical outcomes related to higher satisfaction scores of both YP and parents. In a more recent study, McNicholas, Reulbach [21] did not report a cross-informant effect but found that parent-reported clinical change and parent-reported satisfaction had a significant relationship. Studying the long-term effects of treatment outcomes and satisfaction with CAMHS, Solberg, Larsson [24] and Godley, Fiedler [18] found no association between the reports of YP and parents.

Other shortcomings in the field involve small sample sizes [20, 25] and few validated measures of user satisfaction [23, 26]. It has been suggested that satisfaction should include measurements of the relationship with the clinician, the physical environment and the organization of services [26, 27]. The Experience of Service Questionnaire (ESQ) is a user satisfaction instrument developed for CAMHS [28, 29], with the constructs of general satisfaction (GS), satisfaction with care (SWC) and satisfaction with environment (SWE). However, to our knowledge, only SWC has been studied in relation to outcomes [9].

YP user satisfaction is underrepresented in the research literature; moreover, there is a stronger association between YP satisfaction with clinical outcomes than parent satisfaction [23]. The available research on satisfaction with CAMHS suggests gaps in knowledge. To address these gaps, the aims of this study were twofold: first, we aimed to determine whether the different dimensions of satisfaction, namely, GS, SWC and SWE, of YP and parents predicted clinical outcomes; second, we aimed to determine whether the interaction between YP and parent satisfaction impacted clinical outcomes. In this study, our research questions are primarily exploratory in nature. Given the gaps in the existing literature and the complexity of user satisfaction within the realm of CAMHS, we aim to investigate and uncover potential relationships and patterns. By adopting this exploratory approach, we hope to generate novel insights that can provide a foundation for future hypothesis-driven studies in this domain.

## Methods

### Participants and procedure

The study participants were YP and parents using CAMHS at the University Hospital of North Norway (UNN) between December 2013 and December 2020. At intake (T1) and at the six-month follow-up (T2), responses from YP, parents and clinicians regarding routine outcome measures (ROMs) were collected in a local quality registry following the Snapshot protocol of the Child Outcome Research Consortium (CORC). Further details on the Snapshot approach can be found elsewhere (https://www.corc.uk.net/resource-hub/sending-data-to-corc/; Wolpert et al., 2008; Wolpert et al., 2016).

To be eligible for the current study, YP (age > 11 years) and/or parents (of YP of any age) had to complete the ESQ. During the inclusion period, 3091 YP were referred to the service [30]. The ESQ was completed by 728 individuals (233 YP and 495 parents). Demographic and clinical characteristics can be found in Table 1.

**Table 1:** Demographics and clinical characteristics

	*Mean (n)*	*Sd*
*Parent ESQ*		
General satisfaction	29.27 (466)	5.15
Satisfaction with care	24.05 (466)	4.68
Satisfaction with environment	7.85 (466)	1.56
*Youth ESQ*		
General satisfaction	29.39 (231)	7.33
Satisfaction with care	21.97 (231)	6.06
Satisfaction with environment	7.42 (231)	1.76
*Parent rated mental health*		
Parent SDQ total score T1	16.12 (466)	6.31
*Youth rated mental health*		
Youth SDQ total score T1	16.58 (231)	5.55
*Clinician rated mental health*		
CGAS T1	54.28 (492)	8.12
HoNOSCA total score T1	12.32 (413)	5.07
*Outcome variables*		
ΔParent SDQ total	-4.32 (466)	5.51
ΔYouth SDQ total	-2.29 (231)	5.55
ΔCGAS	9.18(314)	11.39
ΔHoNOSCA total	-4.41 (310)	5.66
*Demographics*		
	*n*	*%*
Gender		
Boy	287	49.8%
Girl	289	50.2%
	*Mean (n)*	*Sd*
Age (years)	11.67 (576)	3.48
Family stress ^a^	2.33 (466)	2.19
Parent mental health ^b^	13.38 (466)	4.87
Social aptitude scale	17.99 (466)	5.26
Parent SDQ Prosocial	7.26 (466)	2.18
Youth SDQ Prosocial	7.69 (231)	1.84

ESQ = The Experience of Service Questionnaire; SDQ = The Strengths and Difficulties Questionnaire, T1 = intake score; Δ = subtracting the T1 (intake) score from the T2 (six-month follow-up) score; CGAS = The Children’s Global Assessment Scale; HoNOSCA = The Health of the Nation Outcome Scales of Children and Adolescents;^*a*^*= The Family Stress Scale;*^*b*^= Everyday Feelings Questionnaire

In addition to ROMs, electronic patient record (EPR) variables describing aspects of YP’s background and mental health were included. YP and parents responded through the Youth-in-Mind-Portal, which (in addition to the ESQ) included the Development and Well-being Assessment (DAWBA) [31] and the Strength and Feelings Questionnaire (SDQ) [31–33]. Clinicians’ reports on the Health of the Nation Outcome Scale for Children and Adolescents (HoNOSCA) and the Children’s Global Assessment Scale (CGAS) were manually entered into the registry. The data protection officer at UNN approved use of the data from the quality register for research purposes.

Missing values were replaced by imputing 20 datasets generated with the fully conditional specification method including all available variables; these datasets were pooled together to form one complete dataset for each sample.

### Measures

#### Satisfaction with service

The Experience of Service Questionnaire (ESQ) is a measure completed by both YP and parents that assesses the perceived quality of the care received as well as the service environment [28]. The ESQ consists of 12 items rated on a four-point scale (1 = not true, 2 = somewhat true, 3 = definitely true, 4 = don’t know). Higher scores indicate a higher degree of satisfaction. Items answered with “don’t know” were not included in the analysis. The ESQ has a general satisfaction (GS) scale that includes all items and has a score range from 0 to 36 [29]. There are two second-order factors, namely, satisfaction with care (SWC) and satisfaction with the environment (SWE). SWC is assessed with items 1–7, 11 and 12, with a score range of 0–27. SWE is assessed with items 8–10, with a score range of 0–9.

Separate English versions of the ESQ exist for children (ages nine to eleven), adolescents (ages twelve to eighteen), and parents of children/adolescents of all ages; all versions are parallel measures of user satisfaction [29]. However, Norwegian translations are only available for the adolescent and parent versions of the ESQ. In this study, we used the Norwegian adolescent version, referred to as YP ESQ, for adolescents aged eleven years or older, and the parent version for parents.

In this study, the YP ESQ factors GS, SWC, and SWE demonstrated Cronbach’s alpha values of 0.91, 0.92, and 0.61, respectively. For the parent ESQ, the corresponding values were 0.92, 0.93, and 0.61. More information on ESQ items is available at https://www.corc.uk.net/outcome-experience-measures/experience-of-service-questionnaire-esq/.

#### Routine outcome measures (ROMs)

The Strengths and Difficulties Questionnaire (SDQ) [32] is a 25-item questionnaire with subscales for emotional problems, peer problems, behavioural problems, hyperactivity, and prosocial behaviour. Each subscale has five items with a three-point scale (Not true = 0, Somewhat true = 1, Certainly true = 2). The subscale scores range of 0–10. Items in the subscales emotional problems, behavioral problems, peer problems and hyperactivity are included in the SDQ total score, with a range from 0 to 40. Measurement invariance analysis of an English and Norwegian sample, showed that the five-factor structure presented the best fit for the data in both samples [34].The Cronbach’s alpha of the SDQ total score has found to be 0.80 [32]. The SDQ has separate versions for parents and adolescents. The psychometric properties of the SDQ have been validated in Norwegian samples [35, 36]. The internal consistency of the parent SDQ total and the SDQ prosocial scale in this study demonstrated Cronbach’s alpha values of 0.78 and 0.75, respectively. The same values for the adolescent version were 0.78 and 0.68. For more information about the SDQ, please visit http://www.sdqinfo.org.

The Children’s Global Assessment Scale (CGAS) is a clinician rating scale of general functioning of children and adolescents, with a range from 100 (superior function) to 1 (needs constant supervision) [37]. The CGAS has been examined in numerous research papers and is frequently utilized to assess severity of mental health problems and outcome [38]. In a study of inter-rater reliability among professionals in Norway’s child and adolescent mental health sector, the CGAS achieved an intra-class correlation coefficient (ICC) of 0.61 [39]. In a cross-national study a similar ICC was found [40]. See https://www.corc.uk.net/outcome-experience-measures/childrens-global-assessment-scale-cgas/ for an overview of CGAS.

The Health of the Nation Outcome Scales of Children and Adolescents (HoNOSCA) is a clinician rating of mental health problems [41]. It consists of 15 scales that are rated from 0 (no problem) to 4 (severe to very severe problem). In this study the first 13 scales were used, and its total score was used to indicate overall severity of mental health problems (range 0–52). HoNOSCA has been evaluated in several studies and has been found to be easy to use, reliable, valid and sensitive to change [39, 40, 42, 43]. In a nationwide study of the interrater reliability of HoNOSCA in Norway, the interclass correlation (ICC) was 0.84 [40],. The HoNOSCA, as used in our study, yielded a Cronbach’s alpha of 0.50. Please visit https://www.corc.uk.net/outcome-experience-measures/health-of-the-nation-outcome-scales-for-children-and-adolescents-honosca/ for further information on HoNOSCA.

### The development and well-being assessment (DAWBA)

The DAWBA is a comprehensive assessment tool that includes a diagnostic interview and several questionnaires, including the Family Stress Scale (FSS), the Everyday Feeling Questionnaire (EFQ), and the Social Aptitudes Scale (SAS). In this study, the online version of the DAWBA was used. For further details on the DAWBA, please visit https://dawba.info/.

The FSS is a 13-item questionnaire evaluating parents’ perceived stress and socioeconomic status [31]. Stressors related to financial difficulties, unemployment, trouble in the neighbourhood, adequacy of their own home regarding the family’s perceived needs, tensions with partner or ex-partner, illness, gambling- alcohol- or drug misuse are included in the questionnaire. Each item is scored in a three-point scale (none/don´t apply = 0, some = 1, or yes, a lot = 2). The FSS total score has a range of 0–26, with high scores indicating a higher level of family stress. In our study, the FSS demonstrated a Cronbach’s alpha of 0.63.

The (EFQ is a 10-item parent rating of psychological distress and well-being [44]. Parent rate their state during the preceding month. Each item has a five-point scale. The five items measuring distress are scored from 0 to 4, while the five well-being items are scored in the reverse order 4 − 0. High scores on the EFQ indicate higher levels of distress and lower levels of well-being. The EFQ is unidimensional [44, 45]. The Cronbach’s alpha for the EFQ in this study was 0.65. For more information on the EFQ, please visit https://youthinmind.info/EFQ/.

The SAS is a 10-item parent-report questionnaire about their children’s social skills [46]Each item is scored on a five-point scale. The sum score of the items is converted to a T-score. The SAS load into a single factor [46]. High scores indicate better social skills. In this study, the Cronbach’s alpha for the SAS was found to be 0.87. For more information about the SAS, please visit https://dawba.info/SAS/.

The duration of the waiting period (hereafter, waiting time) was measured as the days from referral to the first appointment.

### Statistical analysis

Data were analysed using SPSS version 27. The outcome variables ΔSDQ–Parent, ΔSDQ–YP, ΔHoNOSCA, and ΔCGAS were calculated by subtracting the T1 (intake) score from the T2 (six-month follow-up) score. A series of hierarchal regression analyses were conducted to examine the ESQ scales of YP and parents as predictors of outcomes.

In regression models including ΔSDQ–Parent, ΔSDQ–YP, ΔHoNOSCA, and ΔCGAS as dependent variables, the predictors were entered in two steps. In step 1, the independent variables age, gender, SDQ–prosocial behaviour score, SAS score, waiting time, FSS score and EFQ score were entered. In step 2, the ESQ scale scores of GS, SWC, and SWE were entered in separate models.

In the regression models where the interaction between YP and parent ESQ scores was examined as a predictor of outcomes, the predictors were entered in three steps. The two first steps were the same as previously described. In step 3, the interaction terms parent GS × YP GS, parent SWC × YP SWC, and parent SWE × YP SWE were entered in separate regression models.

## Results

The correlations between the clinical outcome and the specific ESQ scales are presented in Table 2. The associations between ΔYP–SDQ total and YP GS (*r* =.17, *p* <.01) and YP SWC (*r* = −.20, *p* <.01) were significant. The correlation between ΔParent–SDQ total and Parent SWC was significant (*r* = −.20, *p* <.01). None of the correlations between parent and YP ESQ subscale scores were significant, as shown in Table 3. The following significant correlations were observed between T1 intake values of the mental health measures and the ESQ factors: parent SDQ prosocial with parent GS (*r* =.13, *p* <.01), SWC (*r* =.12, *p* <.05), and SWE (*r* =.13, *p* <.01); FSS with parent GS (*r* = −.11, *p* <.05), and SWE (*r* =.12, *p* <.05); YP SDQ prosocial with YP GS (*r* =.14, *p* <.05), and YP SWC (*r* =.16, *p* <.05),

**Table 2:** Correlations between outcome variables and user satisfaction

	Experience of Service Questionnaire					
	Parent GS	Parent SWC	Parent SWE	Youth GS	Youth SWC	Youth SWE
*Outcome variables*						
*Δ Youth* SDQ total	0.10	0.08	0.11	− 0.17**	− 0.20**	− 0.04
Δ *Parent* SDQ total	− 0.07	− 0.12*	− 0.07	− 0.14	-14	− 0.09
Δ *CGAS*	0.11	0.11*	0.07	-	-	-
*Δ* HoNOSCA total	0.04	< 0.00	0.10	-	-	-

*p <.05; ** *p* <.01 (two-tailed test); GS = General satisfaction; SWC = Satisfaction with care; SWE = Satisfaction with environment;

**Table 3:** Correlations between parent and youth user satisfaction

	Youth GS	Youth SWC	Youth SWE
Parent GS	0.05	0.08	− 0.05
Parent SWC	0.05	0.08	− 0.07
Parent SWE	0.06	0.07	0.02

GS = General satisfaction; SWC = Satisfaction with care; SWE = Satisfaction with environment

### YP satisfaction as a predictor of outcome

The hierarchical regression models where GS (F (8, 222) = 2.44, *p* =.2, R^2^ = 0.08) and SWC scores (F (8, 222) = 2.80, *p* <.00, R^2^ = 0.09) predicted ΔYP–SDQ total were significant. In the models, GS and SWC scores predicted 4% and 5% of the variance, respectively. The model with SWE score (F (8,222) = 1.33, *p* =.23) as a predictor was nonsignificant. See Table 4 for the results.

**Table 4:** Hierarchal regression models with Δ Youth SDQ total as dependent variable

	GS in step 2.				SWC in step 2.				SWE in step 2.		
	*R* ^*2*^	*Δ R* ^*2*^	*β*		*R* ^*2*^	*Δ R* ^*2*^	*β*		*R* ^*2*^	*Δ R* ^*2*^	*β*
*Step 1. Control variables*	0.04	0.04			0.04	0.04			0.04	0.04	
Age			0.01				< 0.00				0.15
Gender ^a^			0.11				0.11				0.11
Family stress ^b^			-0.03				-0.03				-0.04
Parent mental health c			-0.03				-0.12				-0.09
Youth SDQ Prosocial skills			0.33				0.04				0.01
Social aptitude scale			0.13		.		0.13		.		0.12
Waiting time (days)			0.13				0.02				-<0.00
*Step 2. The ESQ*	0.08*	0.04**	-0.20**		0.09**	0.05**	-0.23**		0.05	< 0.00	-0.79

*p <.05; ** *p* <.01. All β-coefficients were taken from the last step in the regression models. GS = General satisfaction; SWC = Satisfaction with care; SWE = Satisfaction with environment;^a^*Boy = 1, girl = 2;*^b^= The Family Stress Scale;^c^= Everyday Feelings Questionnaire; SDQ = The Strengths and Difficulties Questionnaire

### Parent satisfaction as a predictor of outcome

The results are presented in Table 5. The regression models where GS (F (8, 457) = 2.17, *p* =.30, R^2^ = 0.05) and SWC scores (F (8, 457) = 2.86, *p* <.00, R^2^ = 0.05) predicted ΔParent–SDQ total were significant. GS and SWC scores predicted only 1% of the variance each. The model with SWE score (F (8, 457) =,1.78, *p* =.08) as predictor was nonsignificant.

**Table 5:** Hierarchal regression models with Δ Parent SDQ total as dependent variable

	GS in step 2.				SWC in step 2.				SWE in step 2.		
	*R* ^*2*^	*Δ R* ^*2*^	*β*		*R* ^*2*^	*Δ R* ^*2*^	*β*		*R* ^*2*^	*Δ R* ^*2*^	*β*
*Step 1. Control variables*	0.03	0.03			0.03	0.03			0.03	0.03	
Age			-0.06				-0.06				-0.04
Gender ^a^			0.02				0.02				0.04
Family stress ^b^			-0.17				-0.17				--<0.00
Parent mental health c			-0.04				-0.04				-0.03
Parent SDQ Prosocial skills			0.13*				0.13				0.12
Social aptitude scale			0.05		.		0.05		.		0.04
Waiting time (days)			-0.04				-<0.00				-0.04
*Step 2. The ESQ*	0.04*	0.01*	-0.10*		0.05**	0.02**	-0.14**		0.03	. -<0.00	0.05

*p <.05; ** *p* <.01. All β-coefficients were taken from the last step in the regression models. GS = General satisfaction; SWC = Satisfaction with care; SWE = Satisfaction with environment;^*a*^*Boy = 1, girl = 2;*^*b*^= The Family Stress Scale;^*c*^= Everyday Feelings Questionnaire; SDQ = The Strengths and Difficulties Questionnair

### YP and parent satisfaction as a predictor of clinician-rated outcome

In the regression models with ΔCGAS as a dependent variable, entering the ESQ factors of GS (ΔR^2^ = 0.01, *p* =.23), SWC (ΔR^2^ = 0.01, *p* =.17), and SWE scores (ΔR^2^ < 0.00, *p* =.55) in step 2 did not explain any additional variance. Entering GS (ΔR^2^ < 0.00, *p* =.85), SWC (ΔR^2^ < 0.0, *p* =.62), and SWE scores (ΔR^2^ = 0.10, *p* =.13) in step 2 in the models with ΔHoNOSCA as the dependent variable yielded similar results.

### Interaction between YP and parent satisfaction as a predictor of outcome

Table 6 presents results from the hierarchical regression models with the interactions between the YP and parent satisfaction as predictors of ΔYP–SDQ total. In the models with ΔYP–SDQ total as the outcome measure, the GS YP × parent interaction (F (10, 150) = 3.76, *p* <.00, R^2^ = 0.20) explained 6% (β = 1.91, *p* <.00) of the variance. The model including the SWC YP × parent interaction (F (10, 150) = 3.66, *p* <.00, R^2^ = 0.20) in step 3 was also significant. The SWE YP × parent interaction explained an additional 4% (β = 1.27, *p* =.01) of the variance. In the model with SWE score as the predictor, entering the parent or YP SWE separately (ΔR^2^ = 0.01, *p* =.67; step 2) or jointly (as an interaction term; ΔR^2^ = 0.01, *p* =.12; step 3) did not explain any additional variance in the model.

**Table 6:** Interaction between parent- and youth satisfaction measures in hierarchal regression models with Δ Youth SDQ total as dependent variable

	GS in step 2. and 3.					SWC in step 2. and 3.				SWE in step 2. and 3.		
	*R* ^*2*^	*Δ R* ^*2*^	*β*			*R* ^*2*^	*Δ R* ^*2*^	*β*		*R* ^*2*^	*Δ R* ^*2*^	*β*
*Step 1. Control variables*	0.11*	0.11*				0.11*	0.11*			0.11*	0.11*	
Age			-0.14					-0.15				-0.9
Gender ^a^			0.54**					0.26**				0.23**
Family stress ^b^			-0.01					-0.03				-0.03
Parent mental health c			-0.20*					-0.18*				-0.13
Youth SDQ Prosocial skills			-0.08					-0.07				-0.07
Social aptitude scale			0.19*			.		0.20*		.		0.14
Waiting time (days)			0.02					0.02				-0.02
*Step 2. The ESQ*	0.15**	0.04*				0.16**	0.06**			0.11*	0.01	
Youth			-1.51**					-1.13**				-0.48
Parent			-1.31**					-0.82*				-043
*Step 3. Interaction*	0.20**	0.06**				0.20**	0.04*			0.12*	0.01	
Youth x Parent			1.91**					1.27*				0.66

*p <.05; ** *p* <.01. All β-coefficients were taken from the last step in the regression models. GS = General satisfaction; SWC = Satisfaction with care; SWE = Satisfaction with environment;^*a*^Boy = 1, girl = 2;^*b*^= The Family Stress Scale;^*c*^= Everyday Feelings Questionnaire; SDQ = The Strengths and Difficulties Questionnaire

**Fig. 1:**
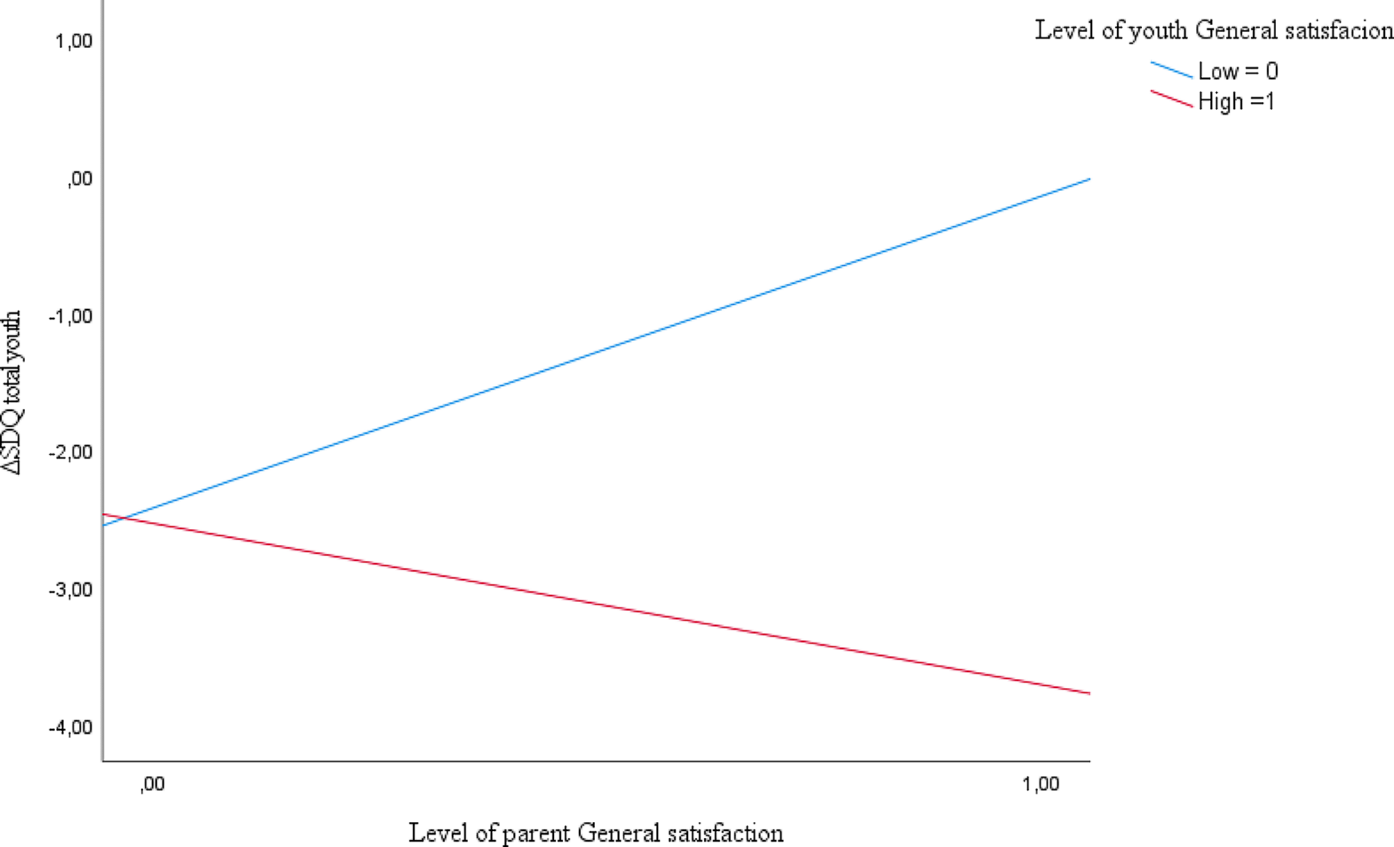
Plot of the interaction between parent- and youth General satisfaction as predictor of Δ Youth SDQ total

To assist with the interpretation of the interaction, ΔYP–SDQ total and GS scores were plotted in Fig. 1. The sample was divided into high and low scores based on the median. High scores in youth and parent GS predicted the best outcome, while the combination of high parent GS and low youth GS predicted the worst outcome. The plot of SWC scores exhibited a similar pattern.

In the hierarchal regression models with ΔParent–SDQ total as the dependent variable, including the YP × parent interaction of GS (F (10, 150) = 1.45, *p* =.04; ΔR^2^ = 0.02, *p* =.09), SWC (F (10, 150) = 2.04, *p* =.03; ΔR^2^ = 0.02, *p* =.08), or SWE scores (F (10, 150) = 1.45, *p* =.16; ΔR^2^ < 0.00, *p* =.86) did not explain any additional variance in the model.

## Discussion

Tracking patient experiences via user satisfaction measures is essential for understanding children and young people’s opinions on care, but it hasn’t been widely adopted despite decades of advocacy [1–3]. User satisfaction, especially within CAMHS, is intricate, with research showing discrepancies between feedback from YP and parents [16–22]. Research on the connection between user satisfaction and clinical outcomes remains sparse [1, 9]. This study explored the associations between different dimensions of YP- and parent-reported user satisfaction and clinical outcomes as well as the interactions between YP and parent user satisfaction as predictors of outcomes. Routinely collected clinical data from CAMHS were analysed. The results showed that both YP- and parent-reported GS and SWC predicted outcomes. The YP and parent interaction of GS × SWC predicted YP-reported outcomes, while the association with the parent-reported outcome was nonsignificant. Similar to most studies [16–22], we found no significant correlations between user satisfaction reported by YP and parents.

For YP, user satisfaction explained 5% of the variance in outcome. For parents, user satisfaction explained 2% of the variance in parent-reported outcomes. In the model including an interaction term of YP GS × parent GS, satisfaction explained 10% of the variance in the YP-reported outcome. Compared to other factors that predict outcomes, such as therapeutic alliance (7%; [47] and psychotherapeutic treatment (13%; [48], this represents a substantial effect. The interaction indicates that low concordance between YP and parent satisfaction was associated with worse YP-reported outcomes. The results emphasize that in a clinical context, dedicating time and effort to improving YP satisfaction with service could be important for their outcomes. Furthermore, the results also suggest that user satisfaction could have a different impact on YP- and parent-reported outcomes.

None of the models with SWE as a predictor of outcomes were significant. This factor comprises items measuring structural and organizational conditions at the service level. The participants in this study were recruited from the same service with common routines. They received mental health services with equivalent service. The participants’ perception of the structural and organizational conditions was the only known factor that could induce variance in SWE. This could have resulted in variation too low to detect any significant associations. SWE is a construct that may be more suitable for between-service comparison than within-service analyses.

In CAMHS, the family unit is often conceptualized as the “patient,” emphasizing the interconnectedness of individual and familial experiences in therapeutic contexts. Within this framework, the concordance between parent- and YP-reported satisfaction becomes especially salient. This alignment can be viewed as an extension of the therapeutic alliance, a factor presumed to be associated with positive treatment outcomes [49]. The therapeutic alliance [50], characterized by a shared understanding of therapeutic goals, agreement on the tasks that constitute therapy, and an emotional connection between the therapist and the family members, may influence the concordance of satisfaction levels between parents and young people. When both parties share similar perceptions and evaluations of the therapeutic process, it may indicate a unified understanding of the therapeutic goals and outcomes within the family. Conversely, discrepancies in satisfaction might hint not only at challenges within the therapeutic relationship but also at potential tensions within the family unit. Recognizing and addressing these discrepancies is pivotal for clinicians as they aim to strengthen the therapeutic alliance and ensure that interventions resonate with the entire family, thereby enhancing the overall efficacy of care in CAMHS.

### Study limitations

The main limitation of this study is that user satisfaction and outcome were measured at the same timepoint. This makes it difficult to determine the causality of relationships between the variables. This concurrent measurement raises questions about bidirectionality: could clinical outcomes or psychopathology levels influence user satisfaction just as satisfaction might impact outcomes? It is possible that better clinical outcomes—indicative of reduced psychopathology—can enhance satisfaction with the services. In the Donabedian model for examining the quality of health care, the categories “structure,” “process,” and “outcomes” are used to operationalize dimensions of quality [51]. In this framework, satisfaction with care represents a process factor yielding information about how health care is delivered, while satisfaction with the environment is a structure factor reflecting the context of health care. In the Donabedian model, it is assumed that structure and process variables facilitate outcomes. The purpose of satisfaction surveys is to capture how the patients and their caregivers perceived mental health care. Methodologically, it is challenging to examine the causality of relationships between satisfaction and outcome with a longitudinal design. However, an RCT in which the intervention group receives an intervention designed to increase user satisfaction could be used to analyse the causal relationship. Alternatively, as in this study, a regression analysis controlling for factors that may influence changes in symptom levels may be conducted.

Another limitation of this study relates to the suboptimal Cronbach’s alpha values observed for some of the measures, with good internal consistency typically represented by a value of > 0.70 [52]. Notably, the HoNOSCA, which was used as a dependent variable, had a Cronbach’s alpha of 0.50. A low value for a dependent variable like HoNOSCA could introduce variability not linked to the predictors, potentially compromising the reliability of the regression outcomes. Additionally, the predictors ESQ factor SWE (0.61), EFQ (0.65) and FSS (0.63) also demonstrated suboptimal internal consistency. In the context of regression analysis, predictors with low internal consistency can introduce noise into the data, potentially weakening the observed relationships and leading to underestimated regression coefficients.

### Recommendations for future research

Our study lacked detailed data on specific types of care, such as medication use or modality of psychological therapy, underscoring the need for future research. The nature of treatment can significantly influence both clinical outcomes and user satisfaction, so exploring how different treatments affect these aspects is important. Understanding such correlations would not only elucidate the dynamics of patient satisfaction but also provide insights to healthcare providers to better tailor interventions. Given the potential variability in satisfaction with treatments, particularly in relation to perceived efficacy, side effects, or patient preferences, it would be relevant future studies to include assessment of treatment modalities to ensure the ongoing optimization of care within CAMHS.

## Conclusion

The findings indicate that for YP, user satisfaction predicts outcomes and that disagreement between YP and parents regarding user satisfaction may have a negative effect on outcomes. There was a negligible correlation between YP- and parent-reported satisfaction factors. The results highlight the importance of collecting both parent and YP data for user satisfaction surveys. Indeed, assuming that parent or YP data can be used as a proxy measure for each other may yield misleading results.

Even if the association between user satisfaction and outcome varies, user satisfaction measures represent an important measurement in their own right. The use of such measures can help to identify gaps in service provision, ensure that services are user centred, and facilitate engagement with mental health services. The use and sharing of user satisfaction may demonstrate an organization’s desire for transparency and engagement in quality improvement. For stakeholders and the public, who fund mental health services by taxes or insurance premiums, user satisfaction may be a central dimension of quality. Together with other quality measures, user satisfaction represents an important aspect of a user-centred service that aspires to meet the needs and preferences of the patients and their families.

## Data Availability

The data that support the findings of this study are available from the corresponding author (Børge Mathiassen, borge.mathiassen@unn.no) but restrictions apply to the availability of these data, which were used under license for the current study, and so are not publicly available. Data are however available from the corresponding author upon reasonable request and with permission of the data protection officer at the University hospital of North-Norway.

## Declarations

Moreover, stringent secure storage protocols were followed, and the data from the quality registry were anonymized before being used in this study. All methods employed in this study strictly adhered to relevant guidelines and regulations and were performed in accordance with the Declaration of Helsinki.

In the context of publication consent, this study did not require informed consent as it utilized health register data. The study received approval from the local data protection officer at the University Hospital of North-Norway, who represents the Norwegian data protection authority within the hospital trust, specifically for publication purposes. The data utilized in the study were originally collected during routine quality assurance procedures at the clinic. Given the nature of this data collection, written consent for publication was deemed unnecessary by the data protection officer, who holds the authority to make such decisions in accordance with ethical guidelines and legal requirements.

## Abbreviations

CAMHS: child and adolescent mental health services
ESQ: Experience of Service Questionnaire
GS: General satisfaction
SWC: satisfaction with care
SWE: satisfaction with environment
SDQ: Strengths and Difficulties Questionnaire
CGAS: Children’s Global Assessment Scale
HoNOSCA: Health of the Nation Outcome Scales for Children and Adolescents
YP: Young people
UNN: University Hospital of North Norway
CORC: Child Outcome Research Consortium
DAWBA: Development and Well-being Assessment
ROM: Routine outcome measures
ICC: Interclass correlation
FSS: The Family Stress Scale
EFQ: The Everyday Feeling Questionnaire
SAS: The Social Aptitudes Scale
